# Thermoplastic Composites and Their Promising Applications in Joining and Repair Composites Structures: A Review

**DOI:** 10.3390/ma13245832

**Published:** 2020-12-21

**Authors:** João Pedro Reis, Marcelo de Moura, Sylwester Samborski

**Affiliations:** 1 INEGI—Instituto de Ciência e Inovação em Engenharia Mecânica e Engenharia Industrial, 4200-465 Porto, Portugal; 2 Departamento de Engenharia Mecânica, Faculdade de Engenharia da Universidade do Porto, 4200-465 Porto, Portugal; mfmoura@fe.up.pt; 3 Department of Applied Mechanics, Lublin University of Technology, Nadbystrzycka 36 St., 20-618 Lublin, Poland; s.samborski@pollub.pl

**Keywords:** thermoplastic composites, fusion bonding, resistance welding, induction welding, ultrasonic welding

## Abstract

Thermoset fiber reinforced composites, widely used in current structural applications, have complex repair procedures and generates significant amounts of scrap due to its recycling difficulties, which does not comply with the most recent environmental restrictions. These disadvantages may be overcome by using a thermoplastic matrix phase, which is very suitable to be joined and repaired by local melting, making the composite material fully recyclable. This work presents a literature review on the joining methods applicable to thermoplastic based composites and their potential applications to be used as repair procedures in structural elements. The effectiveness of selected adhesive and fusion bonding techniques for several thermoplastic composite systems is evaluated by a comparative study based on the joints’ strength and toughness results available in the literature. This work focuses on the three most promising fusion bonding techniques: Resistance welding, induction welding, and ultrasonic welding. The advantages and drawbacks for each one of these processes are discussed, as well as their suitability for several specific structural applications. In addition, several discordant aspects for each welding technique are identified and the corresponding recommendations are discussed. A compilation of analytical models for the mechanisms of heat generation and transient heat transfer modelling is also presented for each fusion bonding process in order to promote their application in numerical modelling.

## 1. Introduction

Most of the fiber reinforced polymers (FRP) used in automotive, aerospace, and renewable energy structures have been based on thermoset matrix fiber-reinforced composites (TSC) over the last few decades. Thermoset matrices became very common due to their higher strength and stiffness comparatively to their metallic counterparts, allowing the structural engineers to create lighter components that are more efficient in terms of operational costs influenced by weight reduction. In addition, excellent adhesion, corrosion resistance, resistance to solvents and corrosives, and resistance to heat are also observed [1]. However, TSC have complex repair procedures and generates significant amounts of scrap due to its recycling difficulties. Considering that the environmental legislation is becoming more and more restrictive all over the world, it is evident that waste management is becoming an important issue. Despite of the current notable research available on composites recycling [2,3,4,5], these processes are susceptible to contamination with impurities and fiber damage due to the extreme applied temperature and pressure, and revealed limited efficiency due to its high operation cost. Furthermore, TSC structures may easily suffer impact damage, causing delaminations—preferentially between differently oriented plies, due to mismatch in bending compliances, which can affect significantly their strength mainly under compressive loading [6,7]. In fact, it is observed that the major weakness of the standard carbon fiber/epoxy composite is related to its poor resistance to delamination due to the brittle nature of the thermoset matrix [8] (owing to its high crosslink density). Therefore, research in composite science have been focused on the production of composite systems with higher matrix toughness [9,10]. In this context, hybridization procedures have been applied with reasonable success by including a tougher phase at the most critical interfaces [11]. This approach was applied by Fernandes et al. [12] and de Moura et al. [13], by including cork plies on a unidirectional carbon fiber-epoxy composite laminate to increase its fracture toughness. It was verified that hybridization is advantageous relative to monolithic carbon-epoxy laminate concerning the observed failure mode, which changed from brittle to very ductile mode. Recent experiments by Inal et al. [14] employing thermoplastic non-woven veils between layers were successfully used to increase interlaminar fracture energy under mode I loading in carbon fiber epoxy laminates. Identical experiments carried out by Sonnenfeld et al. [15] revealed significant increase of the impact damage tolerance of epoxy resin based TSC by inserting a thermoplastic film between the thermoset layers. Luo Yu et al. [16] obtained similar results by introducing a soft epoxy adhesive layer at the interface of bismaleimide matrix based composite laminates to increase interlaminar and intralaminar fracture toughness. This process is known as “interleafing” and has been found to increase significantly the impact resistance in fiber reinforced composites [17,18,19,20]. Alternatively, a novel generation of composite systems have been developed employing tougher and more ductile matrices, where thermoplastic polymers are used to compose the matrix phase [21].

Thermoplastic composite materials (TPC) have shown excellent properties and features to meet the current and future demands currently fulfilled by TSC structures. Recent studies in TPC manufacturing and joining processes has encouraged its application by reducing the production costs and allowing efficient assembly of simple parts to produce large and complex structures [22]. This is a notable development as the high resin melt viscosity of thermoplastic polymers and the fiber constraints limit the production of TPC to fairly simple geometries [23,24], which has been one of the main difficulties so far. In addition, the high melt viscosity hampers the manufacture of continuous fiber TPC, as it builds resistance to impregnate the fiber bundles and wet the fibers. Continuous fiber arrangements are preferred for structural applications since they produce a much higher modulus and strength [25], which is particularly true for TPC as thermoplastic polymers have generally lower strength and stiffness than thermoset ones due to their lower crosslink density [26]. In this context, unidirectional prepreg tapes, consisting of thin sheets of continuous reinforcement fibers impregnated with thermoplastic resin, obtained directly from the fiber roving and thermoplastic melt, should be used to manufacture TPC parts with continuous fiber reinforcement [27]. Filament winding process can also be used to obtain TPC tubes or vessels with continuous fiber reinforcement lay-ups [28]. A significant advantage of TPC is their low water absorption, resulting in very low level of moisture uptake, which means their mechanical properties are less degraded by the formation of voids under hot/wet conditions [22,29,30]. This feature was observed in resistance welded lap shear TPC joints performed by Shi et al. [31] and Rohart et al. [32] with laminates previously exposed to moderate moisture content, which did not show a significantly reduction of lap shear strength comparatively to those previously dried.

The most important advantage of TPC is their ability to melt. This particularity of thermoplastic polymers allows TPC to be “reprocessable”, making them fully recyclable and very suitable to be joined and repaired by local melting and re-consolidation process using fusion bonding techniques [33]. This feature is of high relevance as it fits the current environmental requirements, by which many consider them as the next step in composite science materials [27,34,35]. Fusion bonding is also used to manufacture TPC parts, taking place at several interfaces, namely between different yarns and plies of a laminate [36]. Since polymers are generally poor heat conductors, external volumetric heating such as hot tool or infrared radiation would be a quite slow process creating a large heat affected zone. Therefore, internal heat generation is more suitable for join and repair TPC damaged parts [37].

**Figure 1:**
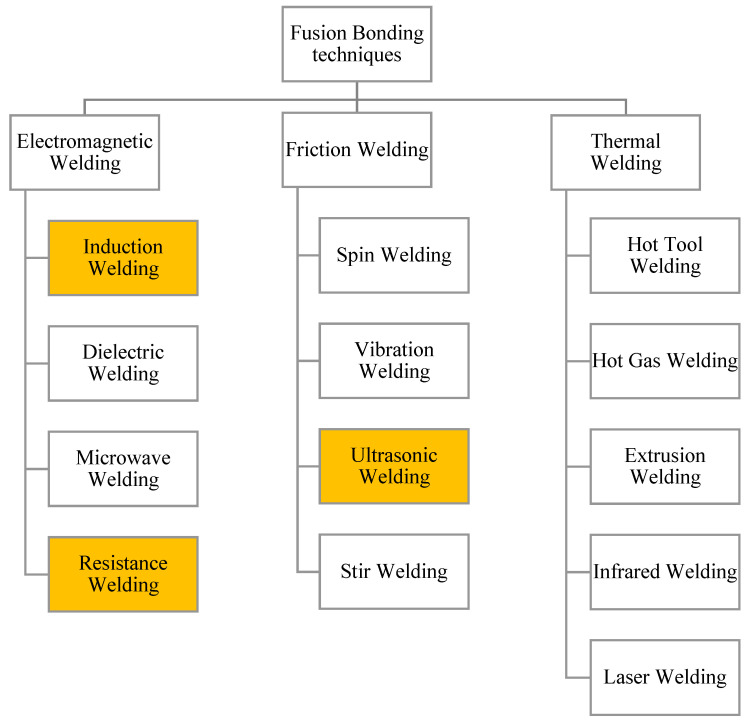
Fusion bonding techniques categorized by the technology used to generate heat. The main fusion bonding techniques are highlighted in yellow.

**Figure 2:**
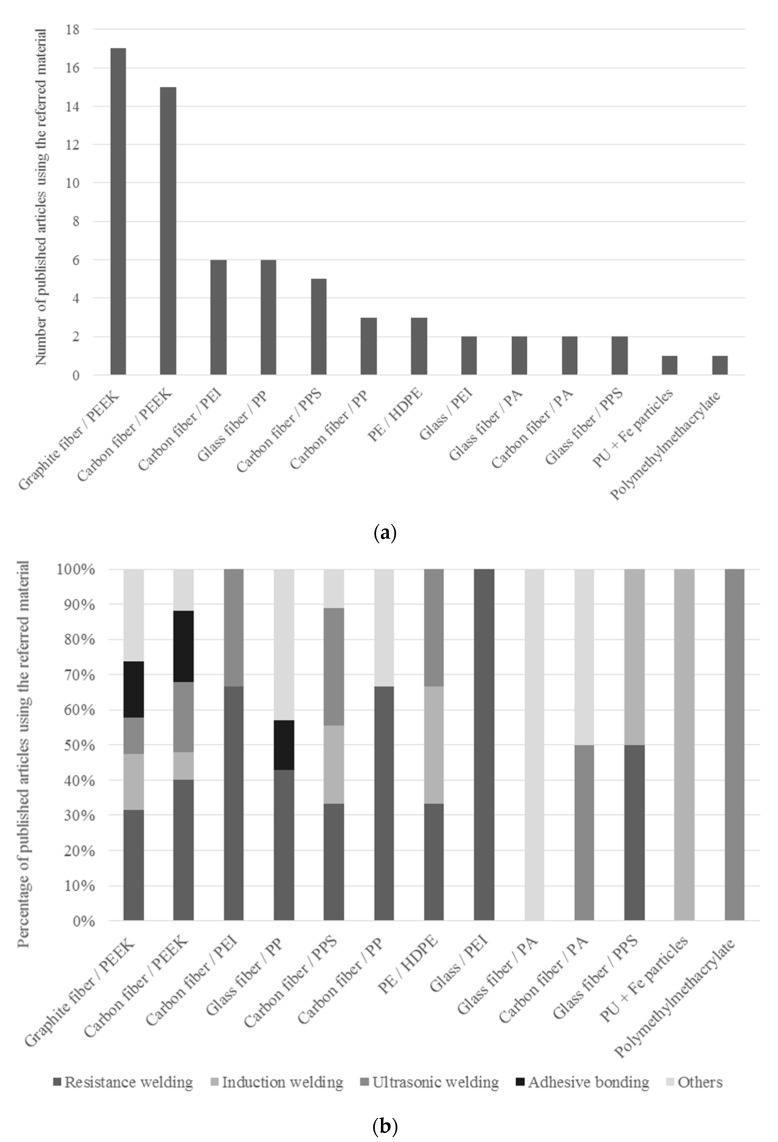
(**a**)—Main thermoplastic composite materials used in fusion bonding research techniques: Graphite fiber/PEEK [58,59,60,61,62,63,64]; Carbon fiber/PEEK [23,47,60,65,66,67,68,69,70,71,72,73,74,75,76,77] Carbon fiber/PEI [69,78,79,80,81,82]; Glass fiber/PP [34,40,83,84,85,86]; Carbon fiber/PPS [29,32,33,48,87]; Carbon fiber/PP [36,70,88]; PE/HDPE [42,89,90]; Glass/PEI [31,80]; Glass fiber/PA [83,85]; Carbon fiber/PA [36,91]; Glass fiber/PPS [92,93]; PU + Fe particles [94]; Polymethylmethacrylate (PMMA) [95]. Keywords of the search scope: “Fusion bonding”, “Resistance welding”; “induction welding”; “ultrasonic welding”. (**b**)—Distribution of manufacture techniques to perform bonded and welded joints on the thermoplastic composite materials presented in Figure 2a.

Fusion bonding techniques are classified according on the heat generation mechanisms on the bonding interface, namely thermal welding, friction welding, and electromagnetic welding (Figure 1). A number of welding techniques are available for each heating mechanism, but the three most promising ones are based on internal heat generation mechanisms, namely resistance welding (RW), induction welding (IW), and ultrasonic welding (UW) [38], which are the ones treated in this work. Extended descriptions about the remaining ones, including the influence of process parameters which mostly affects the welding strength, can be consulted on the literature review on fusion bonding processes by Yousefpour et al. [39]. Several apparatus were initially developed in 1990s aiming to perform welded interfaces mostly in polyether-ether-ketone (PEEK)-, polyetherimide (PEI)-, and polypropylene (PP)-based TPC substrates, by resistance heating (Table 1), in order to study experimentally the influence of the process parameters including pressure, temperature and processing time (Figure 2a,b) [40,41]. Numerical modelling has been also used to perform this task, mainly for resistance welding processes, although most of the recent investigations are still based in experimental trials (Table 1). PEEK polymer showed great potential for aerospace and energy industries for its excellent mechanical, thermal, and chemical properties, which could be further enhanced when reinforced with carbon fibers [23]. More recently, polyamide (PA) and polyphenylene sulfide (PPS) based TPC have also been used to perform welded joints. Carbon fiber has been broadly used as TPC reinforcement to perform fusion bonded joints (Table 1). The referred fusion bonding techniques have been successfully employed in TPC with reasonable results, taking advantage of their higher fracture toughness and better chemical, impact and damage tolerance. Applications include joints in plastic pipes, medical devices, wind turbine blades, and aircraft and boat structures [38,39,42,43,44,45,46]. Large-scale applications have also been successfully implemented using resistance and induction welding with reduced assembly time [47]. Specific examples in aircraft industry include the leading edges of the wings of Airbus A340-600 and A380, which are assembled by resistance welding, and the empennage (tail) of the Gulfstream G650, which is assembled by induction welding [48]. Unlike mechanical fastening, fusion bonding does not induce stress concentrations and fiber breakage caused by the drilling operations, and does not require surface preparation and long curing cycles as adhesive bonding procedures. In addition, considering the current abundant usage of thermoset composites and the benefits of using multi materials within a single composite structure, fusion bonding techniques have also been employed to join thermoset to thermoplastic composites by including a compatible thermoplastic polymer layer in the TSC laminate, known as co-cure method [38,49,50]. Possible thermoplastic candidates are polyetherimide (PEI), polysulfone (PSU), poluethersulfone (PES), and polyamide (PA), which have been used to improve toughness in cured epoxies [51]. The major challenge of this procedure is to avoid thermal degradation of the thermoset polymer under the high temperatures required to achieve fusion bonding of the thermoplastic polymer. An option to minimize this undesirable effect was proposed by Villegas et al. [52,53] reducing the heating time by fractions of a second using ultrasonic welding. Alternatively, a thermoplastic film impregnated with fiber fabric may be used to coat the TSC laminate, in a process called hybrid interlayer method [54]. This method was successfully performed by Ageorges et al. [55] using PEI reinforced with glass fiber to provide mechanical interlocking between the TPC and TSC parts. Alternative methods for joining TSC to TPC parts include surface pretreatment to increase the adhesion of the TSC part. Zhang et al. [56] used a solvent containing dissolved epoxy amine resin to improve the surface adhesion of a PA66 glass fiber composite in order to perform adhesive bonding to a carbon epoxy substrate. The obtained lap shear strength with was increased by more than 3 times when compared with untreated surfaces. Amend et al. [57] demonstrated the capability of laser-based surface treatment to join TPC to TSC parts. This laser-based method was used to remove the thermoset matrix from the fibers, enlarging the effective joining area. The laser ablation creates a rough surface with micro undercuts, which improve the joint strength by providing mechanical interlocking between the joining parts.

A number of literature reviews about fusion bonding techniques for joining and repair TPC structures are available on the literature [33,38,39,58,103], including specific overviews for resistance welding [41] and induction welding [24] with full descriptions of the processes and its advantages. However, the most recent literature review with background on fusion bonding of TPC was published in 2012 by Villegas et al. [33], which is quite old considering the large amount of works published in the last few years. Therefore, and considering the growing visibility of TPC nowadays, an updated literature review including the latest contributions in this field is of great interest to the scientific community who is (and will be) working in joining and repair of TPC structures. The present work reviews in detail the state of the art of TPC fusion bonding technology aiming to provide a deeper insight into the nature of thermoplastic welding processes and the research effort that has been put into it by a large number of researchers in the last few years. The physical mechanism involved in the fusion bonding processes are discussed for modelling purposes including heat transfer, consolidation, and crystallinity aspects. Finally, an overview of the bonding strengths obtained for several thermoplastic composites systems using adhesive and fusion bonding techniques are presented for comparison purposes. Both strength and toughness testing results are used to access the quality of the joints in order to evaluate the advantages and disadvantages of each bonding technique.

## 2. Strength Testing in FRP Joints

### 2.1. Strength Testing

The lap shear test is the most used experimental procedure to evaluate joint efficiency in fiber reinforced polymer materials (FRP), thanks to its simplicity. It might be an interesting method concerning comparative results, where it is possible to verify which material can offer the highest strength under shear loading. This test can be performed using several setups, mainly single lap, double lap, lap-strap, and thick adherend shear tests [104]. The single lap shear is the most popular one, and the failure mechanism is determined by the failure type: Adhesive or substrate failure. However, the average shear stress at rupture is the only provided data, and most real applications are not restricted to shear loading, reducing the accurate applicability of this kind of experiment to characterize real applications. Moreover, lap shear specimens are subjected to large stress concentrations and induces unknown mixed mode I + II loading ratio which varies dramatically along the joint length, thus invalidating a proper joint characterization [105]. Recent experiment trials of fastened and welded joints in TPC conducted by Zhao et al. [106] revealed higher tensile strength of the fastened ones thanks to its reinforcement in the out-of-plane direction, absent on the welded bonded joints. These results show that the lap shear test may be insufficient to evaluate the joint strength in many cases. For the industrial applications, a proper joint strength evaluation should be carefully performed to potentiate the use innovative TPC materials.

### 2.2. Toughness Testing

#### 2.2.1. Pure-Mode Loading

**Figure 3:**
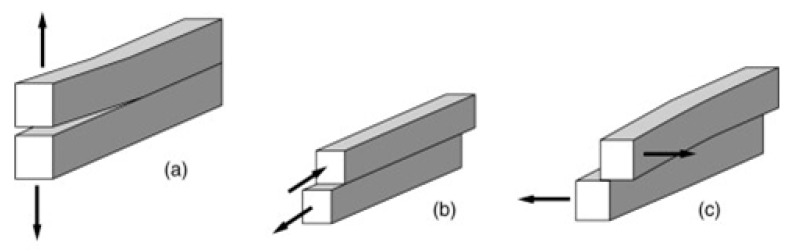
The three loading modes that promote the crack propagation: (**a**)—Mode I (opening), (**b**)—Mode II (in-plane shear), and (**c**)—Mode III (out-of-plane shear).

**Figure 4:**
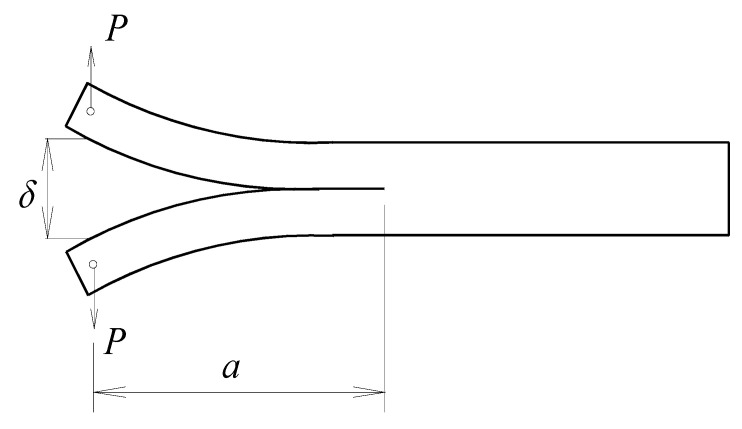
Schematic representation of the double cantilever beam (DCB) test. *P* is the imposed load, *δ* is the crack opening displacement, and *a* is the crack length.

**Figure 5:**
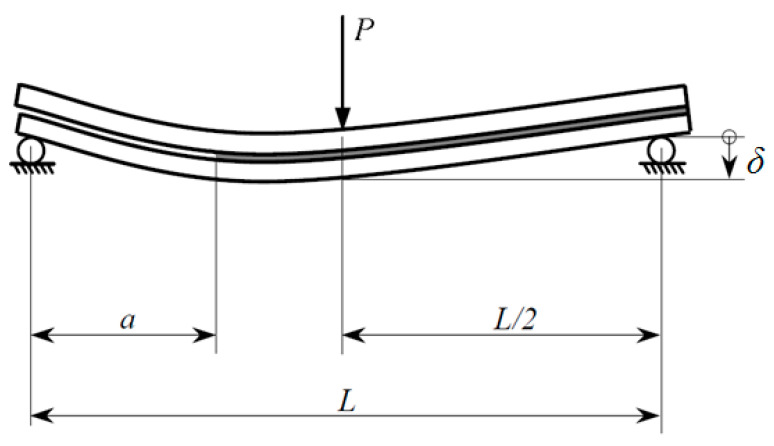
Schematic representation of the End Notched Flexure (ENF) test. *P* is the imposed load, *δ* is the specimen’s deflection, *L* is the length of the specimen, and *a* is the crack length.

The understanding of failure in adhesive and fusion bonded joints is based on mechanical tests that are designed to introduce controlled stresses to the joints. In view of the above mentioned deficiencies of the lap-shear test, pure, and mixed-mode loading toughness tests should be used to evaluate the fracture behavior of a manufactured joint. In pure-mode loading tests the force is applied to simulate exclusively each mode of failure (Figure 3), which is defined according to the loading that promotes the crack propagation [107]:

- Mode I (Figure 3a): The load is applied normally to the bonded joint interface and the rate at which the joint opens can be monitored and measured in the double cantilever beam tests (DCB) (Figure 4). Specimens contain a pre-crack at one of its extremities and the cracked faces are pulled apart with the aid of either piano hinges or loading blocks attached to the specimen on the cracked end. A thin non-stick film is placed between the central plies during curing to introduce the pre-crack, and the sides of the specimen are marked with a millimeter scale in order to quantitatively track the crack growth during testing. The specimen is then loaded and the load-displacement data is recorded and used for computation of the critical strain energy release rate *G*_Ic_. This test can be performed under quasi-static and cyclic loading conditions, and is the most widely used method for measuring Mode I fracture toughness of unidirectional composites. Furthermore, fabrication and testing of DCB specimens is straightforward and relatively inexpensive, which can be tested by using standard mechanical test frames [108];
- Mode II (Figure 3b): The load creates a sliding shear mode in a direction perpendicular to the leading edge of the crack and the joint will exhibit the highest resistance to fracture. The most suitable method to evaluate this failure mode is the End Notched Flexure specimen (ENF) (Figure 5), which consists in a three-point bending test in a pre-cracked specimen. The resulting load creates an almost pure shear stress state at the crack tip, provided that the specimen is designed so that the adherends deform elastically, which provides shear characterization. The simplicity of this specimen is one of the main reasons to be widely used in mode II fracture characterization [109];
- Mode III (Figure 3c): This mode involves a tearing motion or anti-plane shear mode, and does not occur as often as the other two in FRP structural applications, even though it may appear in result of elastic couplings [110,111,112,113].

#### 2.2.2. Mixed-Mode I + II Loading

In practical applications of structural components, the imposed loads result in mixed-mode fracture conditions, which is enhanced by the anisotropy of the composite materials. In fact, many structural applications include single lap joints, which leads to a combination of in-plane tension and shear (modes I and II, respectively). Therefore, fracture characterization under mixed-mode I + II loading tests are essential tasks in order to understand how structures behave in real scenarios. Several approaches have been used to develop test specimens with single and combined normal and shear stresses on the delamination plane. For this reason, mixed-mode fracture tests should always be included in fracture characterization of FRP joints produced both by adhesive and fusion bonding techniques [114].

In cracked lap shear (CLS) test, uniaxial loading is applied to one arm of a split unidirectional laminate [115]. The load transfer to the other arm causes interlaminar normal stresses (mode I) and interlaminar shear stresses (mode II). Although the CLS specimen can be tested in conventional tension testing machines, it has several serious limitations related with large rotations caused by the load eccentricity at the delamination front [116]. In the Arcan test configuration, a split unidirectional laminate is bonded between two metal fixtures that can be loaded to produce various mixed-mode conditions at the delamination front [117]. However, the mode I + II ratio must be determined by a numerical analysis and bond failure can limit its usability, especially for tough laminates, as is the case of TPC. The asymmetric DCB (ADCB) test is a generalization of the standard DCB test, in which different arms thickness is considered. Therefore, in ADCB samples the crack plane is out of the laminate mid-plane inducing a mixed-mode loading state at the crack tip. This test is appropriate for determining the mixed-mode delamination toughness of laminated composites with different mode ratios by varying the thickness of each arm. In this test, the mode II component at the crack tip is limited up to 20% approximately [118]. The Mixed-Mode Bending (MMB) test is a combination of the DCB and ENF tests widely used to characterize the mode I and II, respectively. Consequently, the MMB loading can be represented by a superposition of a pure mode I and pure mode II loading, equivalent to those used in DCB and ENF, respectively. This test works by adding an opening mode loading to an ENF test. The loading distance defines the relative value of the two loads applied to the specimen, and therefore the mode ratio [116]. The relative value of these two applied forces, establishes the mode-mixity degree at the crack tip. It should be noted that these two forces applied to the specimen result from only one loading applied through a loading beam and a hinge. The Single-Leg Bending (SLB) is a standard three-point-bending test on a pre-cracked specimen, similar to the ENF test [119]. The difference relative to the ENF test resides on the specimen’s geometry, which presents one single loaded arm on the extremity containing the pre-crack. Basically, the SLB specimen can be obtained by cutting the lower arm of the ENF specimen, which induces mixed-mode I + II loading on the crack tip with nearly fixed ratio.

## 3. Comparison of TPC and TSC Fracture Toughness

A major obstacle to efficient application of fiber-reinforced composite materials is their tendency to delaminate. The knowledge of delamination growth behavior, which is the most predominant and life-limiting failure mechanism in composite structures, is thus essential for materials development, selection, design and life-prediction studies. Consequently, characterization of improved delamination resistance and, in turn, more damage-tolerant composite structures, both in pure and mixed modes, has been a major goal of composite materials research. In recent years, a common approach to the characterization of delamination growth has been through the application of the linear elastic fracture mechanics, which enable the critical energy release rate or fracture toughness, Gc, to be deduced [120].

### 3.1. Pure Modes

**Figure 6:**
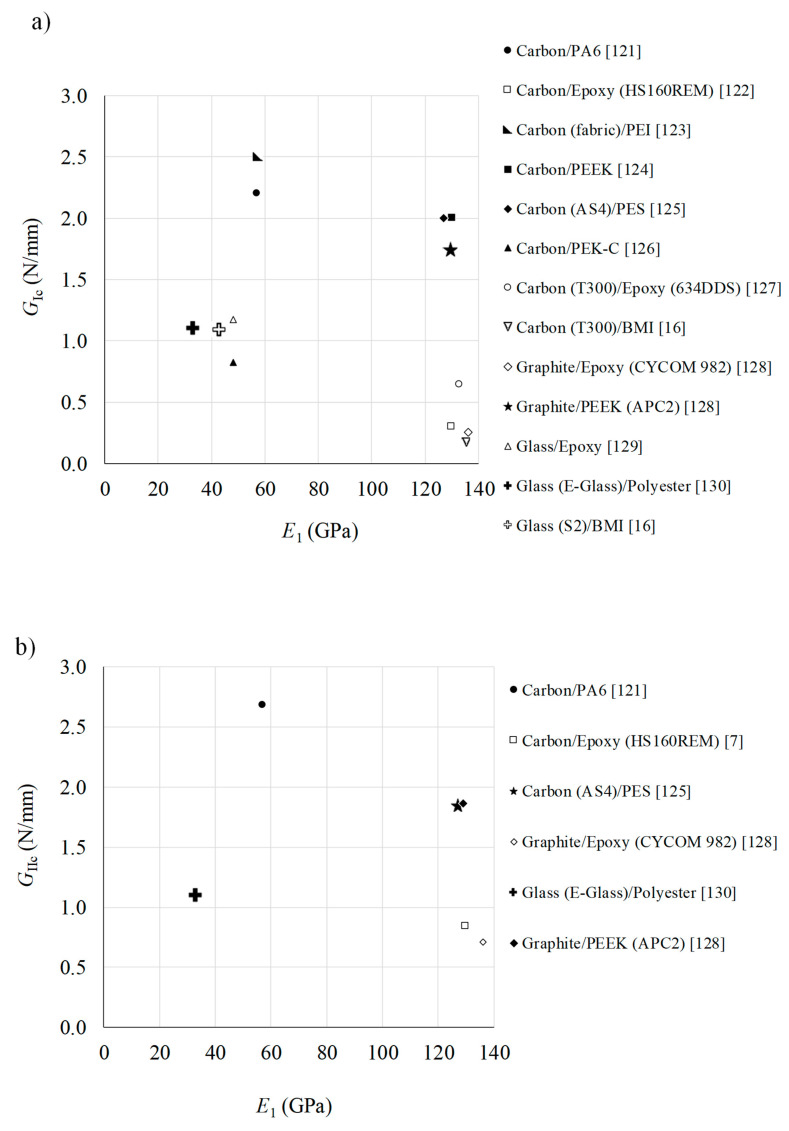
Comparison of critical energy release rates *G*_Ic_—(**a**) and *G*_IIc_—(**b**), and elastic modulus E_1_ of unidirectional thermoset and thermoplastic based composites. Filled and hollow marks stands for thermoplastic and thermoset systems, respectively [7,16,121,122,123,124,125,126,127,128,129,130].

In Figure 6a,b the average interlaminar fracture toughnesses for pure modes I and II loadings are shown: *G*_Ic_ and *G*_IIc_, respectively, for several composite systems picked from the literature review with thermoset and thermoplastic matrices. In order to discuss the relationship between the fracture toughness and the materials’ strength, the elastic modulus in longitudinal direction is plotted with the *G*_Ic_ and *G*_IIc_ values. It can be observed that most of the TPC taken from the literature reveal much higher fracture energies in mode I and mode II when compared to the TSC counterparts, which constitutes a remarkable advantage of the former ones. This is an expected result, since interlaminar toughness of the TPC is known to be higher than the thermoset ones. Although the distribution of the longitudinal modulus reveals lower strength when E-glass fibers are used as reinforcement material, they should not be disregarded for structural applications—their lower cost make them very attractive, and the fracture toughness plays an important role in many FRP applications when impact loading is susceptible to occur. In fact, it is important to note that the modulus and strength of TPC are mainly controlled by the fiber properties, fiber weight fraction and fiber orientation when continuous fiber reinforcement lay-up is used, while the fracture toughness is primarily governed by the matrix properties [25].

### 3.2. Mixed Modes

**Figure 7:**
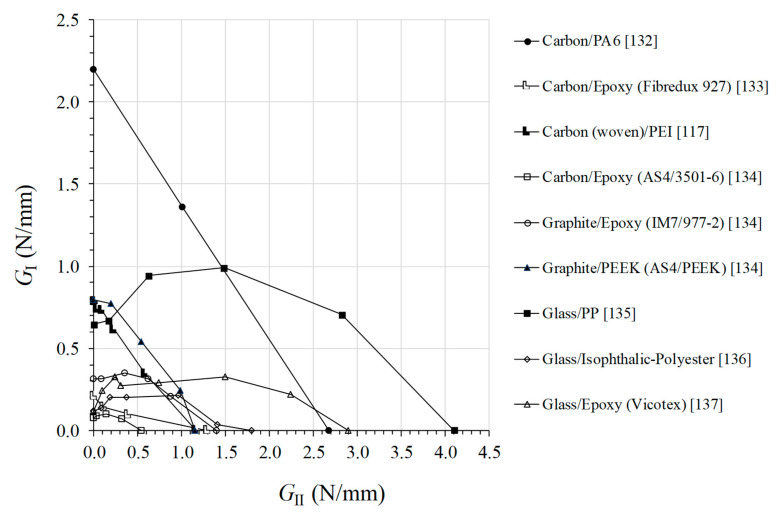
Comparison of critical energy release rate of unidirectional composites under mixed mode loading. Filled and hollow marks stand for thermoplastic and thermoset based composites, respectively [117,132,133,134,135,136,137].

A comparison study between the obtained interlaminar fracture envelopes is shown in Figure 7 for several thermoset and thermoplastic based composites. Different mixed mode ratios are included in order to cover a large range in the *G*_I_–*G*_II_ space. According with the collected data, the thermoplastic based composites presents higher toughness when compared with the thermoset ones, mainly when mode I loading is predominant in the mixed mode ratio. Since the mode I fracture toughness is generally lower than corresponding mode II in most of the thermoset based composite materials, this aspect presents a notable advantage in this context. Therefore, thermoplastic polymers matrices reveals to be promising candidates to replace thermoset based ones in many applications. For example, structural components that are eventually exposed to low velocity impact loading will improve their interlaminar resistance if TPC is employed instead of thermoset based ones. The linear failure criteria can satisfy most of the existing cases displayed in Figure 7. However, some cases reveal an increase of the mode I component with a certain amount of *G*_II_, which decreases after to zero when the applied *G*_II_ equals *G*_IIc_. This behavior is known as “overshoot” phenomenon, and has not been observed for mixed-mode fracture for isotropic materials. Only a few of failure criteria can capture this behavior [131].

## 4. TPC Joining and Repair Techniques

### 4.1. Adhesive Bonded Joints

The adhesive bonding method is based on a patch applied over the substrates on the damaged region similarly to what happens in thermosetting based structures. Apart from the design of the repair patch, the surface treatment and application methods are crucial to its performance. Adhesively bonded patches can be produced using common aerospace epoxy or acrylic based adhesives. Since thermoplastic polymers have lower surface energies when compared to thermoset ones, which makes it difficult for adhesives to wet the adherend surface and create a good bond, correct adhesive choices are therefore critical in order to produce strong and durable bonds [120]. In this context, recent developments in adhesive technology have presented two-part acrylic adhesives designed to improve adhesion in low energy surfaces [138]. Furthermore, surface treatment is critical in any adhesive bonding operation, which is used to clean the contaminants and to improve the wetting of low energy surfaces, chemically modify the surface (introduction of polar groups or coupling agents) and increase the surface roughness (improving mechanical interlocking and increasing bonding surface area). The effectiveness of several surface treatments using epoxy adhesives was studied in [30,62,63,65,66,96,97,98,100,101] by means of DCB and lap-shear tests and Scanning Electron Microscopy (SEM) analysis. Reported methods include solvent wiping, mechanical abrasion, acid etching, grit blasting, plasma etching, flame treatment, and corona discharge treatment. However, surface treatments may be unnecessary if acrylic adhesives are used, which were especially developed for low surface energy materials, such as TPC [138,139]. The investigation about adhesive bonding of thermoplastics is mainly focused on PEEK based reinforced composites, which may not be enough for the current industry applications. Several components in aerospace and automotive applications include PEI, PES, and PPS based reinforced composites [25,27], for which adhesive bonding capability should be addressed.

#### Numerical Modelling of Adhesive Joints

The rapid growth of adhesively bonded joints in structural applications has conducted to the development of models to predict the behaviour of these joints under different types of loading. The combination of the finite element method with the cohesive zone modelling (CZM) has been used with notable success to model adhesive bonded joints in FRP materials [140]. CZM uses a stress-based analysis to model damage initiation and fracture mechanics to deal with damage propagation, allowing one to simulate damage onset and non-self-similar crack growth. Damage propagation takes place without user intervention, does not require the definition of an initial crack, and is usually based on a softening relationship between stresses and relative displacements between crack faces. Cohesive elements, known as interface finite elements, are usually zero thickness elements defined by two surfaces of nodes where each node in one surface is attached to another node on the other surface. These surfaces correspond to the interface plane where the adhesive is placed, which are coincident before the occurrence of any deformation. This way, CZM describes the relationship between stresses and relative displacements of each pair of adjacent nodes [141]. Several types of cohesive models have been developed based on the shape of the interface law, or constitutive law [142]. However, considering that the most of modern adhesives present ductile behavior, trapezoidal CZM are the most adequate laws to simulate accurately such event.

Drawbacks of this approach would be that the crack path must be pre-defined and that damage would be restricted to the path of the cohesive elements [143]. However, in adhesive bonded joints damage propagation is restricted to well defined planes corresponding to the interfaces between adhesive and adherends or inside the adhesive. Therefore, the application of CZM is appropriate to model adhesive bonded joints and has revealed good efficiency to simulate its fracture behavior both in TSC and TPC materials. Other relevant example of CZM application to modelling structures is delamination in laminated composite materials [121,132]. On the other hand, as shown by Samborski et al. [110,111,112,113,144,145] also the virtual crack closure technique (VCCT) implemented in many contemporary FE codes, works well from the point of view of modelling delamination in layered structures, such as the FRPs.

### 4.2. Fusion Bonded Joints

Fusion bonding of TPC involves the application of localized heating to the regions to be bonded in order to form a weld between the surfaces and allowing it to cool down under the application of pressure. The interface to be joined must be heated above the glass transition temperature *T*_g_ for amorphous polymers, and the melting temperature *T*_m_ for semi-crystalline ones, while keeping the maximum temperature below the degradation point of the polymer. Semi-crystalline thermoplastic polymers need higher heating energy to flow because they have orderly molecular arrangements. The heat affected zone strongly affects the quality of the welded joint. In general, a plastic material should not be welded at a temperature above than 75% of its glass transition point *T*_g_ for amorphous polymers, and 75% of its melting point *T*_m_ for semi-crystalline ones [89]. Therefore, the study of temperature distribution is of significant importance to optimize any fusion bonding process. Thermocouples placed between the welding interface may be used to monitor the temperature changes during the experimental trials.

The surfaces to be repaired must first be brought into intimate contact under applied pressure and the temperature at the interface must be raised to allow molecular diffusion across the interface. Little surface preparation beyond cleaning is required, which reduces the execution time [38,39]. Careful must be taken regarding the distortions and residual stresses caused by the local heating to avoid deconsolidation of previously well consolidated areas of the laminate due to the involved melting. In this context, constrained setups must be used to maintain the original shape. These undesirable effects may be mitigated by reducing the proximity of thermal gradients among the different parts to be joined [29,74]. In the case of semi-crystalline substrates, an interlayer of an amorphous polymer may be used to perform a joining process called Thermabond method [47,97]. In this process, also known as dual polymer bonding, a layer of interlayer polymer film is placed in the areas to be bonded of the laminate, prior the consolidation process. During the consolidation, both polymers are melted to allow intermolecular diffusion and to create a bond between them. After cooling, the obtained composite laminate includes a thin layer of the interlayer polymer on the surfaces to be bonded. The interlayer is then heated just above its glass transition point to allow fusion bonding, but well below the melting point of the semi-crystalline polymer. According to Smiley et al. [18,97], the key to successful bonding with this process is to use an interlayer polymer that is molecularly compatible with the reinforced polymer.

Fatigue performance was evaluated by Villegas et al. [33] for ultrasonic, induction and resistance welding of lap shear joints of carbon fiber reinforced PPS. The obtained S-N curves revealed similar for all the three types of fusion bonded joints with infinite fatigue life around 40% of the corresponding static lap shear strength. Similar results were obtained by O’Shaughnessey et al. [48] for static lap shear strength for all the referred fusion bonding processes. Fatigue response of ultrasonically bonded joints of carbon/Elium^®^ thermoplastic composites was recently investigated by [146], revealing an increase of fatigue life by 10–12% when compared to adhesively bonded joints. Fatigue performance on lap shear unidirectional carbon fiber reinforced PEI and PEKK, and weave glass fiber reinforced PEI resistance welded joints was performed by Dubé et al. [80]. Linear S-N curves were observed for all three materials, but infinite life occurred for different percentages of static lap shear strengths for the different TPC materials. Therefore, according with O’Shaughnessey et al. [48], the selection of a fusion bonding process for a particular application should be determined by factors such as the material type, weld size and geometry. However, Davies et al. [66] found that resistance heating provides significant stronger repairs comparatively with ultrasonic heating when using a PEI heating element. Therefore, this aspect is not clear.

There are several fusion bonding techniques depending on the technology used for heat introduction. The three most promising fusion bonding techniques are UW, IW, and RW, described in detail in the following sections.

#### 4.2.1. Ultrasonic Heating

**Figure 8:**
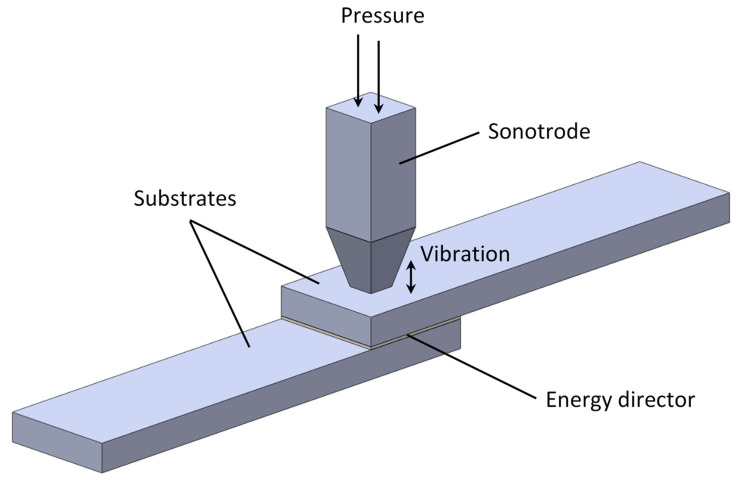
Schematic representation of ultrasonic welding process.

In UW, the parts to be joined are held together under pressure and subjected to ultrasonic vibrations (typically between 20 and 50 kHz), perpendicular to the contact area (Figure 8). The pressure and vibration are applied simultaneously by a sonotrode, connected to a piezoelectric generator, converting high-frequency alternating current into high-frequency mechanical vibrations. The parts are held together by a pressure actuator connected to the ultrasonic welder apparatus. This motion of low and high amplitude oscillations (sinusoidal strain) creates intermolecular friction which is converted to heat. The process can be monitored by the power and displacement data provided by the ultrasonic welder which can be used to follow and identify the different stages occurring at the welding interface. This way, the experimental definition of the optimum processing parameters can be eased for a certain material and welding setup [81]. The relation between the joint strength and each stage of the welding was investigated by Villegas et al. [147]. The strength of ultrasonic welded joints mainly depends on temperature at the interface [89]. Therefore, the main process parameters are the applied pressure, vibration time, solidification (hold) time, and amplitude and frequency of vibration (Table 2) [23,89]. The vibrational energy normally concentrates around the surface’s man-made asperities, called energy directors, which dissipate heat.

Applications and research activities in ultrasonic welding of TPC have been deeply reviewed and conducted by Villegas et al. [33,81,82,87,147,148,149,150,151,152]. The size, shape, and number of energy directors affect the heat generation and resin flow. In this context, flat energy directors developed by Villegas et al. [147,148] were found to efficiently concentrate the heat generation on the welding interface. Nevertheless, Suresh et al. [89] found that triangular energy directors propitiate higher interface welding temperature than semi-circular ones. The former ones concentrate more heating energy because of its small cross-sectional area, which allows a faster initialization of the melting, exhibiting higher ultimate tensile strength. On the other hand, recent experimental work carried out by Goto et al. [91] testing the shear and tensile strength of cross-ply and woven welded laminate joints of carbon fiber reinforced polyamide revealed that flat energy directors made of a neat polymer layer, with the advantage of not requiring to mold resin protrusions on the surfaces, mainly improves the shear strength only. This result is in agreement with the experimental study conducted by Villegas et al. [87] on ultrasonic welded lap shear joints of carbon reinforced PPS, observing that triangular energy directors heat up two times faster approximately, but the measured lap shear strength of specimens using flat energy directors was slightly higher.

UW is a very fast procedure of joining TPC, capable to develop welded joints in less than 5 s, making it appropriate for spot-welding [35], and assembly of micro devices using micro energy directors [95]. In fact, this method is not suitable for repair procedures to large areas (20 cm by 20 cm) since the bond strengths become variable and many charred and partially bonded specimens may result. In addition, ultrasonic and vibration welding equipment are too heavy for practical in-field work. In general, this method is more suitable for joining parts in automotive and aerospace industries, wire biding in electronics, and in the packaging industry for sealing purposes. However, smaller repairs can be performed by drilling out the damaged region, replacing the hole with a plug of thermoplastic resin and using a portable ultrasonic welding unit to consolidate the weld. Nevertheless, efforts have been taken studying alternatives for upscaling this process. Palardy et al. [82] studied the efficiency of thinner flat energy directors, which can be suitable for welding large bonding areas. More recently, Zhao et al. [151] analyzed the mechanical strength of spot welded single lap TPC joints, including single row joints, double row joints and multi row joints, and the experimental results revealed similar load carrying capacity to that of mechanical fastened joints.

Some published works have proven the importance heat uniformity on the quality of ultrasonic welded joints. Tao et al. [23] ultrasonically welded carbon reinforced PEEK lap shear coupons and reported cracks and voids in the matrix-fiber interface caused by the large difference between the thermal expansion coefficients. Experimental trials carried out by Sun et al. [95] in micro assembly revealed the joint strength is enhanced when decreasing the structure size and increasing the distribution size of the micro energy directors, providing extra space for spreading the melted polymer. In addition, the introduction of a neat film resin ensures a proper wetting of the boding parts and improves the mechanical performance of the welded joint. Experimental trials conducted by Sacchetti et al. [75] on ultrasonic welded samples of carbon reinforced PEEK revealed an increase of interlaminar fracture toughness by increasing the amount of extra resin (PEEK film) at the bonding interface. The authors concluded that this improvement was attributed to the development of microscale matrix plastic deformation.

**Figure 9:**
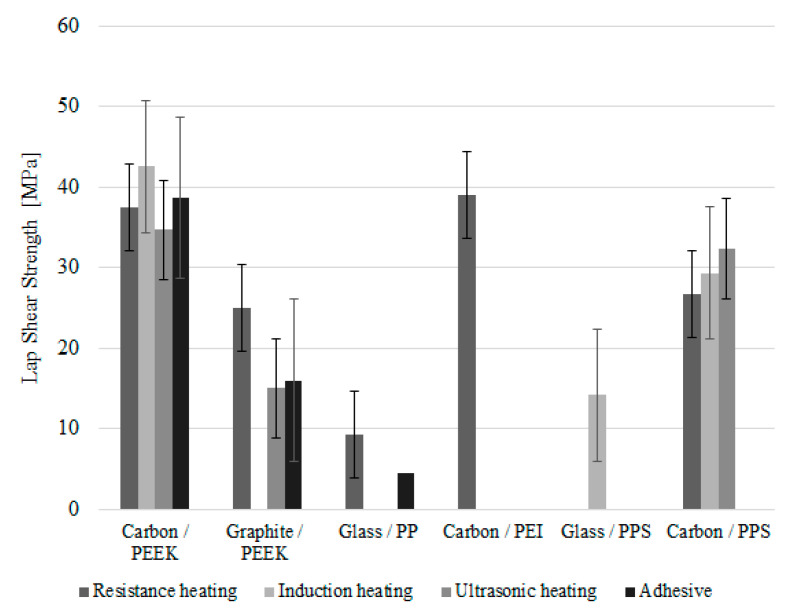
Experimental mean lap shear strength values of several of adhesive bonded and welded joints using thermoplastic based composites substrates.

Several experimental trials using ultrasonic heating were evaluated in several TPCs by [23,33,48,58,63,65,66,87], resulting in lap shear strength values ranging between 15 and 35 MPa (Figure 9).

#### 4.2.2. Induction Heating

**Figure 10:**
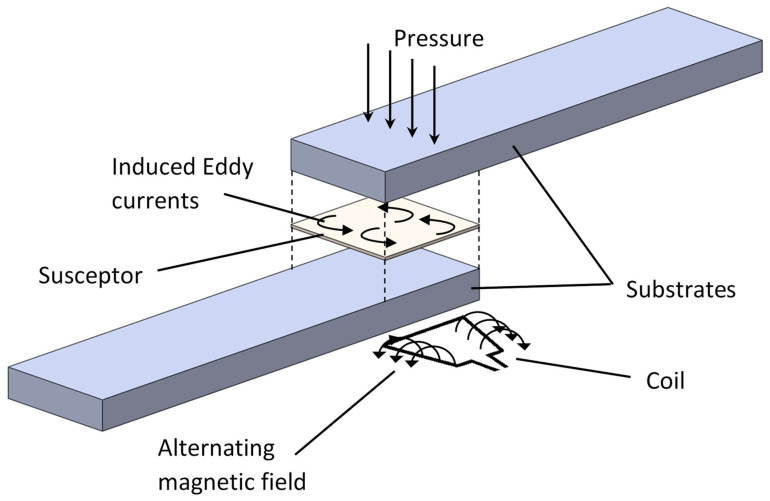
Schematic representation of induction welding process.

IW is a non-contact method of heating thermoplastic composites containing a conducting element. Local heating may be achieved in short times using an induction coil that generates a time variable magnetic field. Eddy currents are induced in the electrical conductor placed in the vicinity of the magnetic field, and heat is generated by Joule losses (Figure 10). This way, high temperatures are easily obtained with a steel mesh screen placed along the bondline. The coil should be placed as close as possible to the part to assure maximum energy transfer. The main process parameters that govern the welding process are the frequency, input power, applied pressure, and the welding time [24] (Table 2). The heating generation also depends on various material properties, such as material type (consolidates or non-consolidated plies), fiber architecture (woven or unidirectional), and lay-up and polymer matrix. [153]. The fact that the conductive element remains in the joint after welding, enables the possibility of reprocessing or repair in the case of incomplete bonding or damage event, respectively [43]. Portable induction welding units are available for field repair operations and provide rapid heating times of less than a minute. When bonding large areas (e.g., 20 cm by 20 cm) the induction heating produces low bond strengths and large variation of bond integrity can be found with some charring of the material. In these cases, continuous IW may be preferable by moving the coil along the joint, which is major advantage of IW process [74].

In composites that use graphite or carbon fibers to reinforce thermoplastic resins, the fibers themselves can act as the conductive element, closed-loop circuits formed by a conductive network of waves or cross plies. Embedded micrometer metallic particles can also be used as conductive elements for heat generation [92,94]. Using the material itself as a susceptor has the advantage of avoiding the use of contaminating inserts on the bondline, which can affect negatively the mechanical performance of the welded joint [24]. Ferromagnetic particles were successfully used by Bayerl et al. [90] as heating promotors to melt thermoplastic polymers within a reasonable time (less than 3 min). However, the influence of particle size and frequency levels are not in agreement considering several works using micrometer metallic particles as susceptor [90,92,94]. In view of the great interest on nanostructured polymers nowadays, induction welding can be potentially used to join nanocomposite parts when the polymer matrix is filled with electrically conductive nanofillers [154].

Once again, the heating uniformity appears to have a significant impact on the induction welded joints. The effect of a stainless-steel mesh based heating element and the input current of the coil on the lap shear strength welded joints was studied by O’Shaughnessey et al. [48] revealing that lower heating rate (around 5.0 °C/s) results in higher lap shear strength since it promotes better temperature homogeneity along the weld interface. However, careful must be taken since very low heating rates leads to undesirable deformation of the adherends due to the excessive temperature increase throughout the thickness. Constrained setups should be used in these cases.

The effectiveness of induction heating in several TPC was evaluated by [33,48,58,60,62,65,74,92,94] with lap shear strength values raging between 14 and 43 MPa (Figure 9).

#### 4.2.3. Resistance Heating

**Figure 11:**
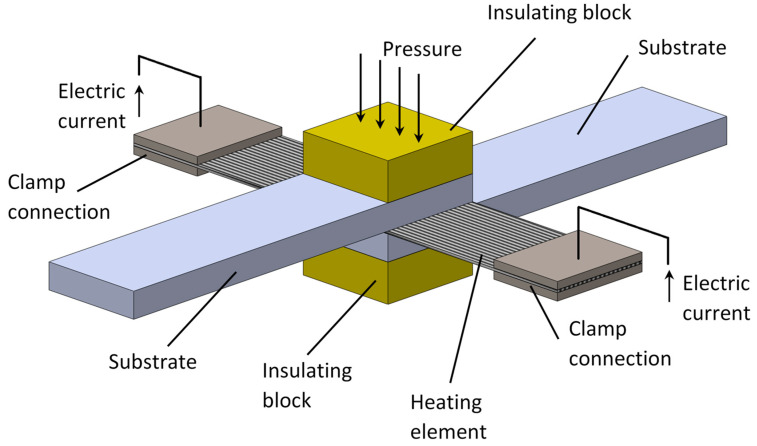
Schematic representation of resistance welding process.

RW process involves trapping a conductive implant between the two parts to be joined. Electrical current is then transmitted through the insert causing the increase of the temperature as result of resistance heating. This way, high temperatures are generated over the bonding interface causing the melting of the thermoplastic polymer (Figure 11). This method presents the advantage of allowing the application of the implant welding to complicated joints, as well as to a more demanding layups, such as those with elastic couplings, where the total number of plies can exceed 30 [110,111,112,113,144,145]. Similarly to the IW method, the conductive implant remains in the joint after welding, which can be useful for further reprocessing operations. However, it may affect the mechanical performance of the bond and increases the weight of the assembly and the risk of corrosion [41]. Nevertheless, this aspect is not clear since some authors claim that it does not affect negatively the mechanical performance of the welded joint [155]. A drawback of resistance welding is that it brings a risk of heat flow disorder (local boiling of the resin) in thicker packages of plies during the autoclaving production process. Under these circumstances, thinner laminates should be joined together to ensure good quality of the test specimens. Initial studies on resistance welding include the influence of mechanical pressure, power density, and fiber architecture on the temperature distribution on the heating element of resistance welded lap shear joints of PEI based TPC, in order to define a processing window [78,79]. According with Panneerselvam et al. [86], the main contributing process parameters for the lap shear strength of resistance welded joints are, by decreasing order of influence, the welding current, welding duration, and clamping pressure (Table 2). In addition, Stavrov et al. [41] includes the resistance of the heating element as also an important process parameter. Prolonged or over heating leads to polymer degradation and promotes undesirable fiber motions due to excessive melting, whereas too much clamping pressure cause expulsion of melted polymer from the interface. On the other hand, increasing the applied pressure minimizes the process induced voids, mainly those caused by fiber de-compaction [156]. Nevertheless, experimental results obtained by Howie et al. [99] showed an inverse relationship between the lap shear strength and the bondline thickness, which is significantly affected by the applied pressure.

Material properties may also affect the strength of the welded joint. Several works reported a reduction in lap shear strength if the fiber orientation of the adherends is perpendicular to the load direction [31,47]. Recent studies about process parameter optimization of resistance welded joints of glass fiber reinforced polypropylene laminates was recently developed by [40,86]. In these works, a quantitative relationship between the joint strength and the pressure, time, and current transmitted to the heating element was established by the Response Surface Optimization Method [40] and Taguchi Method [86].

The resistance heating element must be insulated from any conductive constituents of the composite. The introduction of neat resin film as an interlayer not only serves the diffusion process by creating a resin rich region like in any fusion bonding process, but also provides, in this case, thermal and electrical insulation to the laminate. According with Xiao et al. [67], the insulation of the heating element controls the temperature evolution of the interface between the resin film and the laminate. In fact, prevention of the current leakage was found to improve the temperature homogeneity over the welding area by Dubé et al. [72]. Therefore, it may be considered as a process parameter which affects the strength of the resistance welded joint.

Thermocouples placed along the bonding interface may be used to read the temperature evolution during the welding process. Alternatively, thanks to the relationship between the resistance and the temperature of the heating element, an additional way to monitor the interface temperature can be applied by measuring the current and the voltage of the heating element and following the Ohm law [157]. Alternatively, the process can be monitored by measuring the vertical displacement of the welding stack (i.e., heating element and adherends), which can be related to the physical phenomena taking place along the welding interface, namely welding defects such as voids and resin squeeze flow [158].

When carbon fiber prepreg is used as a conductive element, the ends of a single layer may be treated with chromic-sulfuric acid to remove the resin and expose the fibers, which can be subsequently coated with conductive paint to improve the contact with the connected clamps [67]. However, some difficulties have been reported regarding use of conductive carbon fiber prepregs as heating elements. According with Panneerselvam et al. [86] uneven current distribution and non-uniform heating across the width of the heating element may result in the case of fiber breakage. In such cases, stainless steel mesh is more appropriate. Rzeczkowski et al. [113] reported problematic electrical connections between the electrodes and the carbon fibers, and poor adhesion bonding between the stainless-steel mesh and the polymer, which may decrease the quality of the weld. In addition, the high stiffness of carbon fibers hampers their introduction in complex interface geometries. In this context, an innovative nanocomposite heating element made of PEI with carbon nanotubes (similar to Thermabond process) was recently developed by [76] to weld unidirectional Carbon-fiber/PEEK laminates in single lap configuration. The obtained lap shear strength was not very high—19.6 MPa, but the observations to the fracture surface revealed cohesive failure and non-uniform heating over the weld area. For these reasons, the authors believe this setup can be an interesting alternative to the traditional heating elements if the temperature uniformity could be improved. To overcome this shortcoming, Russello et al. [102] successfully welded thermoplastic PEEK films using embedded layers of Carbon Nanotube web between two PEEK films, obtaining 96% of the strength of the pristine material in mechanical tensile tests. The temperature uniformity was also pointed by Murray et al. [43] to affect the joint strength when welding TPC lap shear coupons by resistance welding. This is in agreement with Eveno et al. [59] which stated that stronger resistance welded joints are obtained with lower power levels that promote temperature uniformity. Ageorges et al. [22] showed that the propagation of the heat generated by the heating element placed between the interface welding surfaces occurs in a much higher rate than the heat in the parts that are exposed to air. This difference is explained by the heating transfer mechanisms associated to each event—conduction between the heating element and the polymer, and radiation and free convection between the external surfaces and the air. The authors believe that this difference, commonly referred as “edge effect”, is responsible for non-uniform heating in many cases, and is influenced by the distance between the electrical connectors and the welding stack, i.e., clamping distance. In this context, Tabolt et al. [73] developed 2D and 3D numerical models to optimize the clamping distance and to avoid polymer degradation at the edges of the weld due to local overheating. For large structural applications, continuous and sequential RW may be used to improve the temperature uniformity along the weld interface [47,159].

The effects of temperature and moisture on carbon fiber reinforced PPS resistance welded lap shear joints was acceded by Rohart et al. [32]. The authors reported 26% and 61% of lap shear strength reduction relative to room temperature by exposing the welded joints at 82 °C and 150 °C (60 °C above *T*_g_), respectively. The moisture did not affect the lap shear strength, thanks to the low moisture uptake of TPC materials.

The effectiveness of this technique in several TPC was evaluated by [32,33,42,47,48,58,59,61,62,63,64,65,66,67,68,69,70,76,78,79,80,84,86,99] and the obtained lap shear strength values bounded between 9 and 37 MPa (Figure 9).

The collected data for lap shear strength of TPC joints (Figure 9), reveals similar lap shear strength for all the three fusion bonding techniques considered in this study. This result is in agreement with the findings reported by O’Shaughnessey [48], where the tested lap shear joints revealed similar strength values for all the three welding techniques. As for adhesive bonded lap shear joints, it should be noted that the presented lap shear strength would be dramatically lower if the adhesive joints were manufactured without any specific surface treatments. Only the best results from each experimental trials are considered in this comparative study for each joining technique and surface treatment.

Nevertheless, considering the above mentioned deficiencies of the lap-shear test regarding with the large stress concentrations and the mixed-mode ratio variation along the joint length, pure loading fracture tests should be performed to evaluate the critical energy release rates (*G*_Ic_ and *G*_IIc_) which measure the fracture toughness of the material. In addition, mixed-mode fracture tests must also be performed in order to evaluate the energetic fracture criterion characterizing the connection. This is an important quality indicator of any adhesive or fusion bonded joint as it quantifies its damage resistance, which is a very important property of FRPs applied in composite structures.

**Figure 12:**
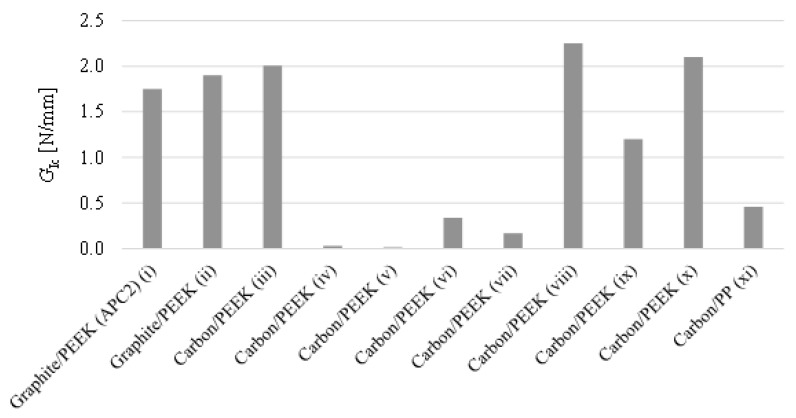
Experimental critical fracture energy release rates (*G*_Ic_) obtained in pure mode I loading fracture tests for several thermoplastic based composites. Legend (joining method, surface treatment): i—Bulk state [128]; ii—Resistance heating [61]; iii—Bulk state [124]; iv—Epoxy adhesive Dexter, abrasion [96]; v—Epoxy adhesive Cyanamid, abrasion [96]; vi—Adhesive Araldite AY103 [66]; vii—Adhesive Araldite AV118(M) [66]; viii—Hot melt adhesive [96]; ix—Ultrasonic heating [66]; x—Resistance heating [66]; xi—Resistance heating [66].

Limited data was found for pure mode I loading fracture tests mainly using carbon/PEEK and graphite/PEEK laminates as shown in Figure 12. This is not an unexpected outcome as these tests are relatively innovative compared to the well-known lap shear tests. In fact, according with Stavrov et al. [41], the DCB test is only used as a supplement test because some works reported it to be unreliable for welded joints as fiber bridging is frequently observed and the crack propagation often occurs within the laminates. As it can be observed, adhesive bonding joints without any other specific surface treatment than abrasion result in very low fracture toughness (low values of *G*_Ic_), which is associated with adhesive failure due to the low energy surface observed in these polymers. On the other hand, fusion bonding techniques revealed much higher values, even exceeding the critical energy release rate of the material in bulk state when resistance heating is used to perform the weld. Contrary with the previous comparison for lap shear strength results (Figure 9), fracture toughness measured by DCB tests points the resistance welding method as the most reliable fusion bonding technique for PEEK based TPC. This result suggests that lap shear test may not give the full picture of the strength of fusion bonded joints.

#### 4.2.4. Non-Destructive Evaluation Techniques

Contrary to metallic structures, the progression of damage in composite applications is still difficult to predict. In particular, for TPC fusion bonded joints, the early detection of small manufacturing defects can affect the load bearing capacity of the welded joints Fusion bonding processes may induce delaminations in fiber reinforced TPC welded parts for several reasons: Expansion of enclosed air, flow of resin caused by excessive pressure, and entrapped air in the interface due to insufficient pressure [160]. In this context, non-destructive testing procedures have been developed to detect delamination zones in TPC welded parts. Taking the advantage of the integrated triangular energy directors for obtaining defective ultrasonic welded joints in a fully controlled way, Ochôa et al. [149] were able to detect unwelded areas and fiber bundle distortion due to overwelding in ultrasonic welded joints by studying the propagation of ultrasonic guided waves. Eveno et al. [59] and Mcknight et al. [47] quantified the quality and extent of resistance welded joints using ultrasonic non-destructive evaluation techniques. Ultrasonic testing was also used by Otheguy et al. [45] to evaluate the extent of the damaged area caused by an impact test on a boat structure made by glass fiber reinforced PP.

However, ultrasonic testing takes long time to scan the welded parts and requires the use of coupling agent. To overtake these disadvantages, induction heating assisted eddy current testing was recently demonstrated by Mizukami et al. [160] as being a suitable process to detect delamination zones in carbon fiber reinforced PPS composite. This procedure is based on the difference of the electrical conductivity between the intact and the delaminated zones, allowing one to detect the damaged regions by high temperature spots.

#### 4.2.5. Numerical Modelling of Fusion Bonded Joints

The modelling of fusion bonding processes includes mainly heat transfer and consolidation phenomena. The objective of heat transfer modelling is to predict the temperature in a welding stack and most of the finite element codes include integrated tools to implement it. Alternatively, it can be manually determined implementing the general heat conduction differential equation that governs the time response of the involved heat flow phenomena. For orthotropic materials, such as composite materials, the general heat equation is defined as follows, for the three-dimensional case:

(1) $$\frac{\partial }{\partial x}\left(k_{x}\left(\frac{\partial T}{\partial x}\right)\right)+\frac{\partial }{\partial y}\left(k_{y}\left(\frac{\partial T}{\partial y}\right)\right)+\frac{\partial }{\partial z}\left(k_{z}\left(\frac{\partial T}{\partial z}\right)\right)+\dot{Q}=\rho C_{p}\frac{\partial T}{\partial t}$$

where *T* is the temperature, *ρ* is the density of the composite, *C_p_* is the specific heat, and *k_i_* and $\frac{\partial T}{\partial i}$ (*i* = *x*,*y*,*z*) are the thermal conductivities and the temperature gradients along the three cartesian directions, which represents the balance of flux of energy through the control volume. The right-hand side of the equation represents the increase of the internal energy per unit of time. The material parameters can be estimated by applying the rule of mixtures be assumed constant within a layer [61]. However, some studies have reported the dependency of electric and thermal materials properties with the temperature, such as electrical conductivity and heat capacity, to have a significant importance in the prediction of the temperature field along the welding interface welding processes [48]. Suitable boundary conditions and initial conditions must be defined for each specific case. The volumetric heat generation term $\dot{Q}$, defined according each fusion bonding technique to account for the rate of heat generation or absorption (Table 3), is defined according with three main mechanism, as follows [61]:

(2) $$\dot{Q}={\dot{Q}}_{gen}-{\dot{Q}}_{melt}+{\dot{Q}}_{crys}$$

where ${\dot{Q}}_{gen}$ is the generated heat (e.g., Joule losses for RW and IW), ${\dot{Q}}_{melt}$ is the heat necessary to promote the melting of the thermoplastic matrix (latent heat), and ${\dot{Q}}_{crys}$ is the heat generated during the crystallization of the thermoplastic matrix.

However, it should be noted that the thermal modelling is not always an indispensable step, as experimental temperature histories, recorded using thermocouples for instance, may be used as input data for a consolidation model. This is of great importance to optimize the welding parameters and the joint strength, preventing laminate deconsolidation and polymer degradation [38,71].

Finite element based numerical models for fusion bonding emerged in the 1990s for resistance welding, where the energy equation was applied to predict the temperature distribution along the substrates [61,67,69]. Finite element software such ANSYS and, more recently, Comsol Multiphysics^®,^ have been successfully used to perform this task [25,89]. Comsol Multiphysics^®^ finite element software is well known for its multiphysics capabilities and have been successfully used to model RW, IW, and UW processes [48]. Since these models are time dependent, they are particularly useful to determine the time to melt the polymer and the time to cause thermal degradation. A micro-mechanics model to investigate the thermal de-consolidation and re-consolidation phenomena was established by Ye et al. [29], allowing one to determine the applied critical pressure at which none of these phenomena takes place.

**Figure 13:**
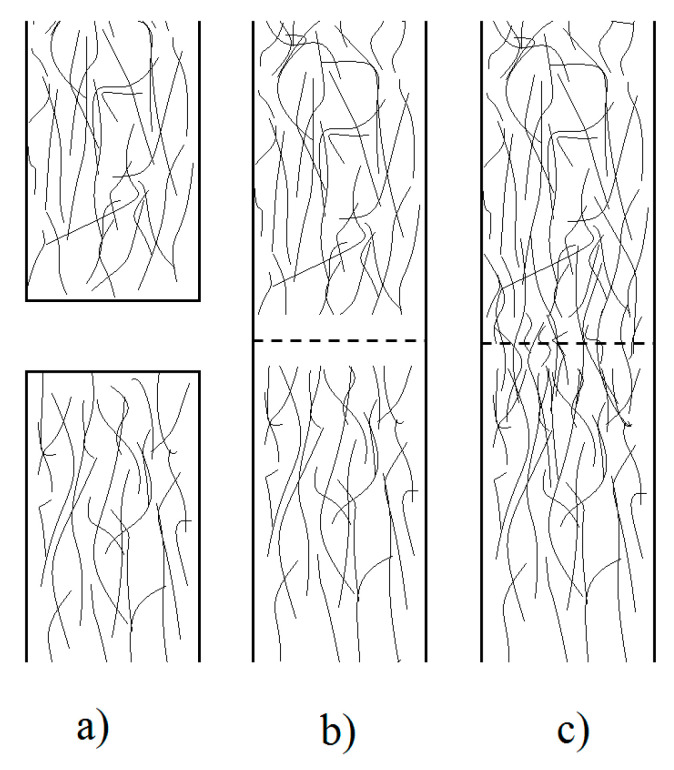
Intimate contact and autohesion stages of the consolidation phenomena: (**a**) Two distinct interfaces; (**b**) achievement of intimate contact; (**c**) collapse of the interface (autohesion).

The consolidation phenomena involves fiber impregnation, resin flow, network deformation, intimate contact and autohesion. The autohesion is a physical phenomenon that describes the diffusion of the polymer chains across the contacted surfaces, i.e., once the surfaces are brought into intimate contact, all the physical barriers existing between the two surfaces disappear and molecules are free to move across the interface (Figure 13) [34,38,51,71]. The intimate contact process depends on the applied pressure, current temperature, surface roughness, and polymer viscosity. These parameters can be related in semi-empirical models, presented by Mantell et al. [163] and implemented by Ageorges et al., Bourban et al. and Shell et al. [34,36,38,71], to determine the degree of intimate contact *D_ic_* at any time during the pressure application *P*, as follows:

(3) $$D_{ic}\left(t\right)=R_{c}{\left[\int_{0}^{t_{p}}\frac{P}{\mu }dt\right]}^{1/5}$$

where *R_c_* is an empirical roughness parameter (see [38] for more details), *t_p_* is the duration of pressure application, and *µ* is the temperature dependent fiber matrix viscosity given by an Arrhenius type correlation:

(4) $$\mu =A\;exp\frac{B}{T}$$

where *T* is the instantaneous temperature and *A* and *B* are empirical determined constants (see [164] for more details). The required time to reach full intimate contact can be reduced by using polymer grades with lower molecular weight. Similarly, the autohesion phenomena can also be described by semi-empirical models to determine the degree of healing *D_h_*, which depends on the instantaneous temperature *T* and the reptation time *t_r_*, which is the necessary time for the polymer chains to diffuse across the interface (i.e., time to get the maximum bond strength). Accordingly, the degree of healing *D_h_* at a time instant *t* can be defined as follows [34,36,165]:

(5) $$D_{h}\left(t\right)={\left(\frac{t}{t_{r}}\right)}^{1/4}$$

Being the reptation time *t_r_* described by an Arrhenius type law:

(6) $$t_{r}=B_{r}\;exp\frac{A_{r}}{T}$$

where *A_r_* and *B_r_* are experimentally determined parameters (see [38] for more details). Since the chains have lower mobility with longer chain lengths, the reptation time *t_r_* increases with the molecular weight. This way, a degree of bonding *D_b_* can be defined as function of the degree of intimate contact and the degree of healing, as follows at a given time *t* [36]:

(7) $$D_{b}\left(t\right)=D_{ic}\left(t\right)\cdot D_{h}\left(t\right)$$

Which expresses the development of the bond strength at any instant of the fusion bonding process and can be enhanced by using polymers with lower molecular weight. In the case of perfect intimate contact, the bond strength attains its maximum value after the elapsed reptation time *t_r_* and the development of the instantaneous mechanical strength *σ* can be described as follows:

(8) $$D_{b}\left(t\right)={\left(\frac{t}{t_{r}}\right)}^{1/4}=\frac{\sigma }{\sigma_{\infty }}$$

where $\sigma_{\infty }$ is the mechanical strength for infinite welding time. Similarly, the bond strength can also be related with the fracture toughness *G_c_* at a given time as follows [165]:

(9) $${\left(\frac{t}{t_{r}}\right)}^{1/2}=\frac{G_{c}}{G_{c\;\infty }}$$

where $G_{c\;\infty }$ is the fracture toughness for infinite welding time. This formulation was applied by Levy et al. [150] while developing a multi-physical model for the heating phenomena in ultrasonic welding with flat energy directors. The temperature profile along the interface and across the adherends was predicted, and the calculated degree of bonding revealed that the adhesion starts at the edges of the interface. This model was later improved by Palardy et al. [152] to account with the hammering effect, resulting from the occasional contact loss between the sonotrode and the adherends due to the high frequency vibration, which directly affects the heating efficiency. Further numerical simulations on UW were conducted by Levy et al. [150,162] to investigate the displacement and adhesion of the traditional energy directors and flat energy directors. However, they require complex numerical methods involving multiphysical formulation of mechanical viscoelastic behavior, flow, and heating factors.

A number of studies have been performed on numerical modelling of IW process [24]. Due to the complex structure of TPC, particular attention has been given how the workpiece generates heat when subjected to an electromagnetic field. In general, three heating mechanisms are considered for heating generation: Joule heating by eddy currents traveling along the conductive fibers; Joule heating by contacting fibers at the junctions (i.e., where fibers from adjacent plies overlap); and heating by dielectric hysteresis when the fibers are separated by a small gap of dielectric polymer matrix. However, it is not clear which one is the dominate heat generation mechanism. Miller et al. [166] and Rudolf et al. [161] performed experimental and numerical studies on induction heating of carbon fiber reinforced TPC and pointed the fiber Joule heating along the fibers and the junctions as the dominant heat generation mechanism, excluding the dielectric heating. However, Flink et al. [167] also performed experimental and numerical studies on carbon fiber reinforced TPC and concluded that the dielectric heating in the matrix region between separated fibers is the dominate heat generation mechanism. Similar work conducted by Yarlagadda et al. [153] identified junction heating by dielectric hysteresis and fiber contacts as the most dominant heating mechanism. This aspect still remains unclear at the present. A full 3D numerical model was developed by O’Shaughnessey et al. [48] for induction heating welding process. The eddy currents distribution was predicted in the heating element and adherends by solving the electromagnetism equations. Subsequently, a transient heat transfer thermal analysis was performed to estimate the heat generated by the eddy currents (Joule losses), and the temperature distribution along the heating element and the adherends as a function of time. The heat transfer in fusion bonding by induction heating was also modelled by Suwanwatana et al. [92], which allow one to predict the transient temperature profile from the coil characteristics and the size of the magnetic particles of the interlayer. Continuous induction welding was successfully modelled by Lionetto et al. [74] using a three-dimensional finite element model to study the heat transfer phenomena, and melting and crystallization in the welding interface. The multiphysical formulation was implemented by coupling electromagnetic and heat transfer equations, and the calculated temperature showed good agreement with the experimental measurements. Afterwards, the numerical model was used to define a process window for the coil speed and coil current to ensure polymer melting and avoid degradation of the matrix.

As for RW process, several numerical works have been presented to study the transient heat transfer and temperature distribution from the heating element. Xiao et al. [67] developed a numerical model using ANSYS finite element software to study the effect of several process parameters, such as input power and fiber orientation, in order to improve the lap shear strength of the resistance welded joints. Ageorges et al. [69,70,71] presented a 3D finite element model to study heat conduction on lap shear welded joints including heat losses by radiation and natural convection. Later, Ageorges et al. [55] developed a transient 3D numerical model for thermal analysis of TPC/TSC welded by resistance heating, using the model to predict the optimal resistance welding time to achieve the maximum lap shear strength. Talbot et al. [73] investigated the effect of the length of the exposed areas of the heating element to air (clamping distance) on the local overheating by using a 2D transient heat transfer finite element model. More recently, in view to upscale RW process, Shi et al. [159] presented a 3D finite element model using COMSOL Multiphysics for continuous RW. The authors divided the process into an electrical model and a thermal model. The first one was used to model the resistance heat generation rate ate the welding interface, while the second one was used to model transient heat transfer over the single lap joint. The welding temperatures predicted by the model showed good agreement with the experimental results.

## 5. Conclusions

This bibliographic review looks at the state of the art of adhesive and fusion bonding technology, focusing particularly on the three most promising fusion bonding techniques: Ultrasonic welding, induction welding and resistance welding. Fusion bonding processes offer additional advantages including reduced surface preparation, reprocessing and recyclability as it promotes the use of thermoplastic composite materials (TPC) to manufacture fiber reinforced polymer (FRP) structures. Comparing with the adhesive bonding methods, fusion bonding techniques present a remarkable potential for volume intensive applications in which short processing cycles are required. The viability of these techniques has been evaluated in terms of the joint strength determined by several authors to join and repair thermoplastic based composites. A small number of researchers have used pure mode loading fracture tests to access the quality of the manufactured joints. In fact, DCB test has only been used as a supplement test because some works reported it to be unreliable for welded joints as fiber bridging is frequently observed and the crack propagation often occurs within the laminates. Therefore, limited data is available using fracture toughness tests of fusion bonded TPC joints.

Several discordant aspects were identified in this literature review:

- It is not clear if different fusion bonding techniques provide different weld strengths for the same substrate. Some authors observed similar weld strength for a TPC laminate using different fusion bonding techniques (RW, IW, and UW), claiming that the selection of a fusion bonding process for a particular application should be determined by other factors such as the material type, weld size and geometry. However, some published works reported RW providing stronger repairs than UW;
- It is not clear if the conductive implant remaining inside the part affects negatively the mechanical performance of RW and IW welded joints. This is an importance aspect since the presence of the conductive implant may be the useful for further reprocessing operations; and
- Is it not clear which one of the three heating mechanisms in IW is the dominant one: Joule heating by eddy currents traveling along the conductive fibers, Joule heating by contacting fibers at the junctions (i.e., where fibers from adjacent plies overlap), or heating by dielectric hysteresis when the fibers are separated by a small gap of dielectric polymer matrix. A deeper insight on IW modelling may be required to clarify this aspect.

Therefore, future work should be carried out to evaluate the quality of the manufactured joints using fracture toughness tests, both in pure and mixed modes, to understand their behavior in real case scenarios. In fact, as suggested by Figure 11 and Figure 12, lap shear tests may not give the full picture of fusion bonded joints. While lap shear test results reveals similar strength values for RW, IW and UW, fracture toughness results for pure mode I loading obtained by the DCB test show higher critical energy release rate *G*_ic_ for RW welded joints. Moreover, the influence of environmental conditions on static and fatigue behavior should also be addressed by submitting specimens to adverse and representative temperature and moisture conditions, to which TPC can be submitted in structural applications. However, it should be noted that not one joining technology can be applicable to all cases. In fact, all joining methods present advantages and drawbacks, and they may be more or less suitable to a particular application depending on its specific requirements.

The modelling of fusion bonding is another critical aspect in joining manufacture processes. The simulations results have established boundaries for the processing windows by accessing the influence of the processing parameters on the quality of the welded joint. Experimental testing is still required, at least to verify model simulations, but is kept to a minimum. Even so, it can be assumed that the modelling of fusion bonding process has not yet fully matured and more work have to be done to achieve a deeper understanding of the several heating methods of fusion bonding techniques. More specifically, a heat-transfer model should be developed to predict the temperature profile over the interface, in order to determine the degrees of intimate contact and healing which fully characterizes the process. These two parameters can be used to determine the interfacial bond strength, and, consequently, to develop an optimization procedure for the process parameters. This way, a relationship can be established between the process parameters and the obtained mechanical properties of the joint, which may be used as input data when modelling the cohesive interface of the manufactured joint.

## Figures and Tables

**Table 1:** Chronological representation of TPC materials applied in Resistance welding (RW), Induction welding (IW), Ultrasonic welding (UW), and Adhesive Bonding (AB) procedures. Gr—Graphite fiber; Gl—Glass fiber, CF—Carbon fiber. Highlighted references represents works including numerical modelling.

Year	RW	IW	UW	AB
1988	Gr/PEEK [58,59]	Gr/PEEK [58]	Gr/PEEK [58]	CF/PEEK [96]
1989	Gr/PEEK [63]; PE [42]		Gr/PEEK [63]	Gr/PEEK [63]
1990	Gr/PEEK [61,62,64]; CF/PEEK [65]	Gr/PEEK [62]; CF/PEEK [65]	CF/PEEK [65]	CF/PEEK [65]
1991	CF/PEEK [66]		CF/PEEK [66]	PEEK [97]; CF/PEEK [66]; Gr/PEEK [98]
1992	CF/PEEK [67]; CF/PP [88]; Gl/PP [84]			
1993	Gr/PSU [99]			CF/PEEK [100]; Gl/PP [100]
1996	CF/PEEK [68]			
1997	CF/PEEK [47]			
1998	CF/PEEK [69,70]; CF/PEI [69,70]			
1999	CF/PEI [78]			
2000	CF/PEI [79]; Gl/PEI [79]			
2006		Gl/PPS [92]		
2007			HDPE [89]; ABS [89]	
2008	CF/PEI [80]; CF/PEKK [72,80]; Gl/PEI [80]			
2011	CF/PEEK [73]			
2012	Gl/PP [86]	HDPE [90]; PA6 [90]	PMMA [95]	PP [101]
2013	CF/PPS [33]; Gl/PEI [31]	CF/PPS [33]	CF/PPS [33]; CF/PEI [81]	
2015		Pu + Fe particles [94]		
2016	CF/PPS [48]	CF/PPS [48]	CF/PPS [48]	
2017			CF/PPS [87]; CF/PEI [82]	
2018	Gl/PPS [93]		CF/PEEK [75]	
2019	CF/PEEK [76]; Gl/Ellium^®^ [43]	Gl/Ellium^®^ [43]; CF/PEEK [74]	CF/PA6 [91]; CF/PEEK [23]	
2020	Gl/PP [40]; PEEK [102]; CF/PPS [32]			

**Table 2:** Process parameters and its typical values of resistance welding (RW), induction welding (IW), and ultrasonic welding (UW).

Process	Heating Time [s]	Process Parameters	Typical Values	Influence
RW	30–300	Power input (kW/m^2^)	30–160	Determines the energy input into the weld
		Welding pressure (MPa)	0.4–1.4	Provide intimate contact and prevent delamination of the heated affected zones
		Clamping pressure (MPa)	4–20	Promotes the lowest resistance on the electrical contact
		Resistance of the heating element (Ω)	$R=\gamma \frac{L}{W}$	Influences the heat generation L—length of the heating element; W—width of the heating element; γ—specific resistance of the material
IW	10–360	Power input (kW/m^2^)		Determines the energy input into the weld
		Welding pressure (MPa)	0.8	Provide intimate contact and prevent delamination of the heated affected zones
		Frequency (Hz)	60–100	Affects quadratically the heating generation
UW	3–4	Power input (kW/m^2^)	80	Determines the energy input into the weld
		Welding pressure (MPa)	2.2	Affects the heating generation
		Frequency (Hz)	20–50	Affects quadratically the heating generation
		Vibration amplitude (µm)	50–85	Affects the heating generation

**Table 3:** Definition of the heat generation term.

Process	Reference	Heat Generation Rate (W/m^3^)	Heat Absorption Rate (W/m^3^)	Parameters
RW	[61,67,73,159]	${\dot{Q}}_{gen}=\frac{I^{2}R}{V}$ ${\dot{Q}}_{crys}=X_{mr}H_{f}\rho \frac{dX_{vc}}{dt}$	${\dot{Q}}_{melt}=X_{mr}H_{f}\rho X_{vc}\frac{dX_{f}}{dt}$ where $\frac{dX_{f}}{dt}=K_{0}exp\left(\frac{-E_{a}}{GT}\right){\left(1-X_{f}\right)}^{n}$	*I*—applied current (A) *V*—volume of the heating element (m^3^) *R*—resistance of the heating element (Ω) *f*—frequency of the coil (Hz) *µ*—magnetic permeability of the composite *H*—magnetic field intensity (Wb) *A*—cross sectional area of the conductive loop (m^2^) *R*_f_—electrical resistance of the conductive fibers (Ω) *H_f_*—enthalpy of fusion (J/kg) *X_mr_*—mass fraction of matrix *X_vci_*—initial crystallinity of the composite *X_j_*—degree of melting *E_a_*—activation energy (J/mol) *K_0_*—pre-exponential factor *n*—kinetic order *G* = gas constant (8.314 J/mol.K)
IW	[24,74,161]	${\dot{Q}}_{gen}=\frac{4\pi^{2}f^{2}\mu^{2}H^{2}A^{2}}{R_{f}}$ ${\dot{Q}}_{crys}=X_{mr}H_{f}\rho \frac{dX_{vc}}{dt}$		
UW	[89,162]	${\dot{Q}}_{gen}=\frac{E’’\omega }{2}\varepsilon^{2}$		*E*’’—loss modulus (MPa) *ε*—amplitude strain (mm) *ω*—vibration frequency of the sonotrode (Hz) *α*_h_—empirical hammering correction factor (0 < *α*_h_ < 1)
	[150]	${\dot{Q}}_{gen}=\alpha_{h}^{2}\frac{E’’\omega }{2}\varepsilon^{2}$		
